# Outcomes of covered stents versus bare-metal stents for subclavian artery occlusive disease

**DOI:** 10.3389/fcvm.2023.1194043

**Published:** 2023-07-07

**Authors:** Libing Wei, Xixiang Gao, Zhu Tong, Shijun Cui, Lianrui Guo, Yongquan Gu

**Affiliations:** Department of Vascular Surgery, Xuanwu Hospital and Institute of Vascular Surgery, Capital Medical University, Beijing, China

**Keywords:** subclavian artery occlusive disease, subclavian steal syndrome, bare-metal stent, covered stent, primary patency

## Abstract

**Objective:**

To compare the clinical efficacy of covered stents and bare-metal stents in the endovascular treatment of subclavian artery occlusive disease.

**Methods:**

Between January 2014 and December 2020, 161 patients (112 males) underwent stenting of left subclavian arteries; CSs were implanted in 55 patients (34.2%) and BMSs in 106 (65.8%). Thirty-day outcomes, mid-term patency, and follow-up results were analyzed with Kaplan-Meier curves. Relevant clinical, anatomical, and procedural factors were evaluated for their association with patency in the two groups using Cox proportional hazards regression.

**Results:**

Mean follow-up was 45 ± 18 months. The primary patency was 93.8% (95% CI, 81.9%–98.0%) in the covered stent group and 73.7% (95% CI, 63.2%–81.6%; *P* = 0.010) in the bare-metal stent group. The primary patency in the total occlusion subcategory was significant in favor of CS (93.3%, 95% CI, 61.26%–99.0%) compared with BMS (42.3%, 95% CI, 22.9%–60.5%; *P* = 0.005). Cox proportional hazards regression indicated that the use of BMSs [hazard ratio (HR), 4.90; 95% CI, 1.47–16.31; *P* = 0.010] and total occlusive lesions (HR, 7.03; 95% CI, 3.02–16.34; *P* < 0.001) were negative predictors of patency, and the vessel diameter (HR, 3.17; 95% CI, 1.04–9.71; *P* = 0.043)) was a positive predictor of patency.

**Conclusion:**

Compared with bare stents, covered stents have a higher midterm primary patency in the treatment of subclavian artery occlusive disease.

## 1. Introduction

Severe stenosis or total occlusion of the subclavian artery (SCA) or the innominate artery proximal to the orifice of the vertebral artery is supposed to cause subclavian steal syndrome (SSS), which makes the blood flow of the ipsilateral vertebral artery reverse into the affected upper limb, resulting in a series of symptoms such as vertebrobasilar insufficiency and subacute or chronic ischemia of the affected limb (1). The clinical symptoms of SSS are related to the rate and degree of arterial occlusion, the compensation of collateral circulation, and systemic blood pressure (2). In addition to the presence of symptoms, mortality due to the disease itself and atherosclerosis-related vascular diseases will increase (3, 4). The treatment of SSS mainly includes medication, SCA bypass or transposition, and endovascular treatment. Endovascular SCA angioplasty with stenting is the first choice for SSS because of its safety, minimal invasiveness, and effectiveness (5). After endovascular procedures, in-stent restenosis is the most common long-term complication, with an incidence of about 8%–15% (6). Many studies on in-stent restenosis of the SCA analyzed the corresponding risk factors, including smoking, diabetes, homocysteine concentration, stent length, blood lipid level, postoperative antiplatelet therapy, and others (7). However, there are few reports about the effect of the stent type (covered or bare) on in-stent restenosis. Currently, the most commonly used stents are bare-metal stents (BMSs), which are prone to induce diffuse intimal hyperplasia and finally lead to in-stent restenosis. Compared with BMSs, covered stents (CSs) may effectively reduce diffuse intimal hyperplasia, which is expected to improve the long-term results (8). The aim of this study was to compare the outcomes in patients with symptomatic subclavian artery severe stenosis or total occlusion who underwent endovascular management.

## 2. Materials and methods

### 2.1. Subjects

The study was approved by our institutional ethics review board. From January 2014 to December 2020, consecutive patients who underwent stenting for de-novo arteriosclerotic occlusive disease of the left SCA at our department were retrospectively analysed. The patients enrolled in this study met the following criteria: 1. Etiology considered to be arteriosclerosis; 2. Degree of SCA stenosis meeting the following requirements: (a) Color duplex ultrasonography (CDUS) or computed tomography angiography (CTA) / digital subtraction angiography (DSA) showing left SCA stenosis ≥ 70% or occlusion; (b) Reverse vertebral artery flow confirmed by imaging examination; (c) Lesion located at the segment of the SCA proximal to the vertebral orifice; (d) Vertebral artery orifice not involved in the lesion. During this time, 106 patients treated with BMSs and 55 with CSs were enrolled, respectively.

### 2.2. Endovascular intervention

After retrograde common femoral artery access with an 8F sheath was obtained, heparin was administered according to the standard protocol (80 U/kg). Angiography was performed to confirm either severe stenosis or total occlusion at the origin of the SCA with reverse flow of the vertebral artery. Brachial artery access was adopted if the guidewire failed to cross the lesion progradely through the femoral access. Brachial access was used in 22 (51.2%) total occlusive patients (14 in the BMS group and 8 in the CS group). After crossing the lesion with a guidewire in total occlusive and extremely severe stenosis cases, pre-dilation were performed with a balloon 3 mm in diameter. Either a BMS (Express LD, Boston scientific) or a CS (Viabahn, WL Gore and Associates) was accurately deployed to cover the lesion. Post-deployment balloon dilatation was performed according to the diameter of the normal vessel distal to the lesion in all the CSs. The femoral accesses were sealed with Proglide (Abbott) and the brachial accesses were compressed manually. Antiplatelet agents and statins were prescribed at the time of diagnosis. Dual antiplatelet therapy (aspirin 100 mg QD and clopidogrel 75 mg QD) were given from at least 5 days before to 3 months after the intervention; lifelong maintenance therapy with either aspirin or clopidogrel was prescribed thereafter.

The CSs were used in cases that there was extravasation of contrast agent after pre-dilation. In other cases, the choice was based on the surgeon's personal preference.

The forward flow of the vertebral artery was confirmed by angiography during the intervention in all the patients. CDUS and upper extremity blood pressure measurements were obtained during follow-up. Primary patency was defined as no evidence of restenosis ≥ 50% or total occlusion within the target lesion, based on CDUS, with a peak systolic velocity ratio (PSVR) ≥ 2.0. The severity of restenosis was evaluated independently by two vascular sonographers who were blinded to the stent type. Disagreements were resolved by a senior vascular sonographer.

### 2.3. Statistical analysis

Data were analyzed using the SPSS 22.0 statistical software. Categoric variables were analyzed using the chi-square test or Fisher's exact test. Mean and standard deviation of continuous variables between the two groups were compared by *t*-test. Kaplan-Meier survival curves were estimated for primary patency and the log-rank *P* value was used to compare two procedures. Cox proportional hazards regression were assessed to determine the association of relevant clinical, anatomical, and procedural factors within the two groups. *P*-values < 0.05 were considered statistically significant.

## 3. Results

### 3.1. Demographic and clinical characteristics

Overall, 161 patients (112 males) underwent left subclavian artery stenting and matched the inclusion criteria. Among those, 55 (34.2%) were treated with CSs, and 106 (65.8%) were treated with BMSs. Demographics, cardiovascular risk factors were similar between the CS and BMS groups (Table 1). There were 45 symptomatic patients (81.8%) in the CS group and 91 (85.8%) in the BMS group. The other 25 asymptomatic patients (10 in CS group and 15 in BMS group) were treated because of planned coronary artery bypass grafting using the internal mammary artery, ipsilateral haemodialysis access, or significant bilateral subclavian stenosis for adequate blood pressure surveillance. The anatomical characteristics, (e.g., lesion length, vessel diameter) were similar between CS and BMS groups (Table 2).

**Table 1:** Patient demographic characteristics.

Characteristic	Overall (*n* = 161)			Stenosis (*n* = 118)			Occlusion (*n* = 43)		
	CS (*n* = 55)	BMS (*n* = 106)	*P*	CS (*n* = 40)	BMS (*n* = 78)	*P*	CS (*n* = 15)	BMS (*n* = 28)	*P*
Demographics									
Age (years)	62.1 ± 6.6	63.4 ± 8.2	0.27	63.2 ± 7.3	63.6 ± 7.9	0.78	59.2 ± 2.9	63.0 ± 9.2	0.13
Male gender	40 (72.7)	72 (67.9)	0.59	30 (75)	52 (66.7)	0.68	10 (66.7)	20 (71.4)	1.00
Cardiovascular risk factor									
Hypertension	26 (47.3)	55 (51.9)	0.62	22 (55.0)	51 (65.4)	0.32	13 (86.7)	18 (64.3)	0.16
Diabetes	15 (27.3)	27 (25.5)	0.85	11 (27.5)	19 (24.4)	0.82	4 (26.7)	8 (28.6)	1.00
Smoking*	18 (32.7)	32 (30.2)	0.86	13 (32.5)	25 (32.1)	1.00	5 (33.3)	7 (25.0)	0.72
Coronary artery disease	13 (23.6)	26 (24.5)	1.00	9 (22.5)	20 (25.6)	0.82	4 (26.7)	6 (21.4)	0.72
Renal insufficiency	2 (3.6)	6 (5.7)	0.72	2 (5.0)	4 (5.1)	1.00	0	2 (7.1)	–
Hyperlipidemia	26 (47.3)	55 (51.9)	0.62	18 (45.0)	42 (53.8)	0.44	8 (53.3)	13 (46.4)	0.75
Medical therapy (before admission)									
None	3 (9.4)	7 (10.4)	1.00	4 (10.0)	8 (10.3)	1.00	2 (13.3)	4 (14.3)	1.00
Antiplatelet	26 (81.3)	54 (80.6)	0.94	34 (85.0)	58 (74.4)	0.24	11 (73.3)	24 (85.7)	0.42
Dual antiplatelet	2 (3.6)	4 (3.8)	1.00	0	4 (5.1)	–	2 (13.3)	0	–
Statins	32 (58.2)	68 (64.2)	0.50	20 (50.0)	48 (61.5)	0.24	12 (80.0)	20 (71.4)	0.72

Data are presented as mean ± standard deviation or as number (%).

* Includes current and former smokers.

**Table 2:** Clinical symptom and anatomical data.

	Overall (*n* = 161)			Stenosis (*n* = 118)			Occlusion (*n* = 43)		
	CS (*n* = 55)	BMS (*n* = 106)	*P*	CS (*n* = 40)	BMS (*n* = 78)	*P*	CS (*n* = 15)	BMS (*n* = 28)	*P*
**Symptoms**	46 (83.6)	84 (79.3)	0.54	33 (79.5)	62 (82.5)	0.81	13 (86.7)	22 (78.6)	0.69
Upper limb ischemia	28 (49.1)	52 (50.9)	0.87	21 (52.5)	36 (46.2)	0.56	7 (46.7)	16 (57.1)	0.54
Vertigo	27 (49.1)	64 (60.4)	0.18	21 (52.5)	51 (65.4)	0.23	6 (40.0)	13 (46.4)	0.76
Ataxia	4 (7.3)	5 (4.7)	0.49	2 (5.0)	3 (3.9)	1.00	2 (13.3)	2 (7.1)	0.60
Syncope	1 (1.8)	1 (0.9)	1.00	1 (2.5)	0	–	0	1 (3.6)	–
Anatomical data									
Lesion length (mm)	21.4 ± 4.5	20.5 ± 4.8	0.24	21.1 ± 5.0	20.2 ± 5.2	0.37	22.1 ± 2.6	21.2 ± 3.2	0.34
Vessel diameter (mm)	7.7 ± 0.4	7.7 ± 0.3	0.63	7.8 ± 0.4	7.7 ± 0.4	0.54	7.6 ± 0.3	7.6 ± 0.2	0.89

Data are presented as mean ± standard deviation or as number (%).

### 3.2. Early results within 30 days after operation

After operation, the pressure difference between bilateral upper limbs was significantly reduced, and the symptoms of most patients were relieved. There were 1 case of ipsilateral axillary artery embolism in the CS group which was treated by surgical-open embolectomy, and 4 cases (1 in CS group and 3 in BMS group) of hematoma at the puncture site (Table 3). There was no stroke, major cardiac event, death, dialysis, wound infection and artery rupture in both groups.

**Table 3:** Early results within 30 days after operation.

	Overall (*n* = 161)			Stenosis (*n* = 118)			Occlusion (*n* = 43)		
	CS (*n* = 55)	BMS (*n* = 106)	*P*	CS (*n* = 40)	BMS (*n* = 78)	*P*	CS (*n* = 15)	BMS (*n* = 28)	*P*
Pressure difference									
Before (mmHg)	34 ± 10	33 ± 13	0.69	33 ± 11	30 ± 10	0.25	37 ± 8	40 ± 18	0.41
After (mmHg)	4 ± 3	5 ± 3	0.80	4 ± 3	4 ± 3	0.46	5 ± 3	5 ± 3	0.46
**Symptom relief**	47 (85.5)	94 (88.7)	0.32	32 (80.0)	68 (87.2)	0.42	15 (100)	26 (93.3)	0.53
Complication									
Stroke	0	0	–	0	0	–	0	0	–
Major cardiac events	0	0	–	0	0	–	0	0	–
Death	0	0	–	0	0	–	0	0	–
Ipsilateral limb artery embolism	1 (1.8)	0	–	0	0	–	1 (6.7)	0	–
Hematoma	1 (1.8)	3 (2.8)	1.00	0	2 (2.6)	–	1 (6.7)	1 (3.6)	1.00
Wound infection	0	0	–	0	0	–	0	0	–
Artery rupture	0	0	–	0	0	–	0	0	–

Data are presented as mean ± standard deviation or as number (%).

### 3.3. Postoperative follow-up

Mean follow-up considering all patients was 45 ± 18 months. Fourteen patients (9.1%) were lost (5 cases in the CS group and 9 in the BMS group) during follow-up. All the lost patients were followed up for at least two years and 6 of them were followed up for more than 4 years.

The mean follow-up time for the BMS and CS groups were 45 ± 18 months and 46 ± 20 months, respectively. In the BMS group, restenosis occurred in 25/97 patients (25.8%), among which 12 cases (12.4%) underwent balloon angioplasty, 5 (5.2%) underwent stent implantation, and 8 asymptomatic cases (8.2%) were treated conservatively. In the CS group, one case (1.8%) died of myocardial infarction one year after the operation. Restenosis occurred in 3/50 patients (6.0%). One patient developed in-stent stenosis 18 months after the operation, and the other two patients developed in-stent occlusion 24 months after the operation. However, the three patients were asymptomatic and were treated conservatively.

**Figure 1:**
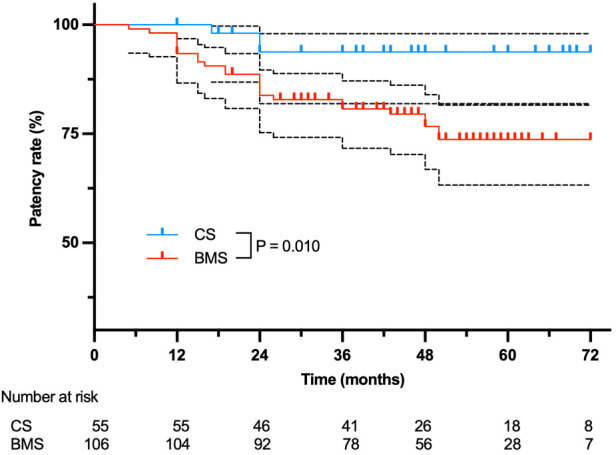
Overall primary patency for 99 patients treated with BMSs or CSs. The black dashed lines stand for 95%CI.

**Figure 2:**
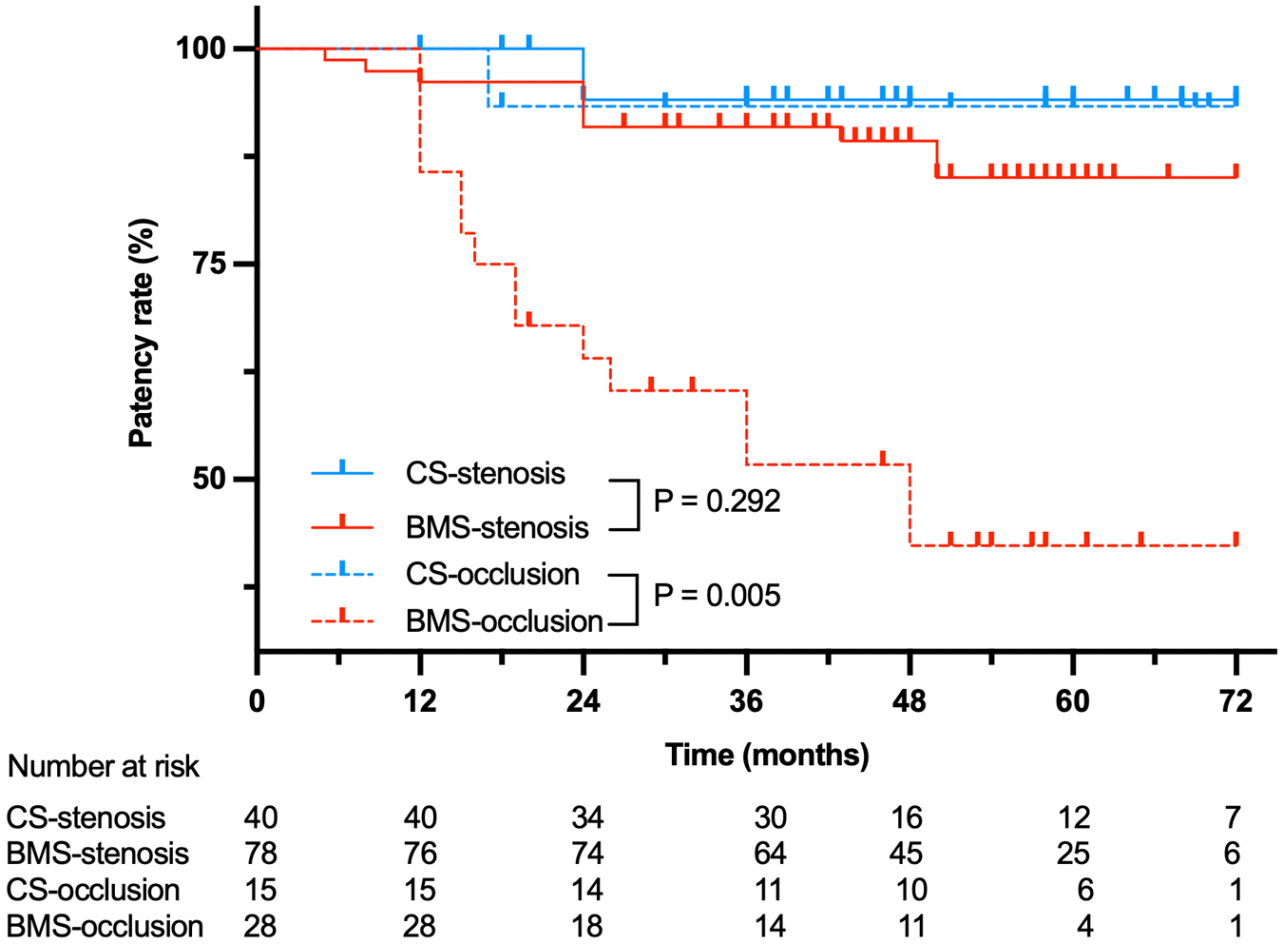
Primary patency for 99 patients treated with BMSs or CSs stratified by stenosis or occlusion.

The 6-year cumulative primary patency was 93.8% (95% CI, 81.9%—98.0%) vs. 73.7% (95% CI, 63.2%–81.6%; *P* = 0.010) for CSs versus BMSs, respectively (*P* = 0.010) (Figure 1). In particular, no significant difference was found in the stenosis subcategory (CS, 94.1% (95% CI, 78.47%–98.5%); BMS, 85.1% (95% CI, 73.5%–91.83%); *P* = 0.292), whereas primary patency in the total occlusion subcategory was significant in favor of CS (93.3%, 95% CI, 61.26%–99.0%) compared with BMS (42.3%, 95% CI, 22.9%–60.5%; *P* = 0.005; Figure 2). Interestingly, the Cox proportional hazards regression showed the use of BMSs [hazard ratio (HR), 4.90; 95% CI, 1.47–16.31; *P* = 0.010] and total occlusive lesions (HR, 7.03; 95% CI, 3.02–16.34; *P* < 0.001) were negative predictors of patency, and the vessel diameter (HR, 3.17; 95% CI, 1.04–9.71; *P* = 0.043) was a positive predictor of patency.

## 4. Discussion

CSs were predominantly used in aortoiliac artery and femoral artery diseases, and led to a better primary patency than BMSs (8, 9). However, there are fewer literatures on the comparison of the efficacy of CSs and BMSs for SCA occlusive disease. This is the first study to compare the midterm results of CSs versus BMSs in patients with left SCA occlusive disease. In this study, the two groups were followed up for 45 ± 18 months. The 6-year cumulative primary patency of the BMS group was 73.7%, while that of the CS group was 93.8% (*P* = 0.010). Besides other risk factors, the type of stent (CSs or BMSs) is an important factor affecting the patency, because the traditional BMS is prone to diffuse intimal hyperplasia leading to in-stent restenosis. Compared with BMS, CS guarantees a mechanical barrier to intimal hyperplasia and also allows aggressive dilatation of calcified vessels, which is expected to improve the long-term patency (8).

The main complication after SCA stenting is in-stent restenosis. There are many reports about the restenosis rate of SCA stenting. Japanese scholars determined a primary patency at 5 years of 80.5% (10). Patel et al. analyzed 170 cases of SSS during 13 years, reporting a patency of 93% at one year and 84% at five years (11). In a retrospective study by De Vries et al., 110 patients with SCA occlusive disease who underwent percutaneous transluminal angioplasty were analyzed (12). The patency of patients with SCA stenosis and total occlusion were 93% and 65%, respectively, three years after treatment, and the difference between the two groups was statistically significant (*P* < 0.05). In this study, we considered 118 cases with left SCA severe stenosis and 43 with left SCA total occlusion. The primary patency at 1 and 5 years of BMSs was 93.4 and 73.7%, which is comparative to the aforementioned studies. The 6-year cumulative primary patency in BMS group for severe stenosis and total occlusion lesions was 85.1% and 42.3%, respectively. In comparison, the primary patency in the CS group for severe stenosis and total occlusive lesions was 94.1% and 93.3%. The primary patency in the total occlusion subcategory was significant in favor of CS over BMS (*P* = 0.005). Furthermore, the result obtained from Cox proportional hazards regression analysis that the use of BMS and occlusion lesions are strong negative predictors of patency may corroborate the concept of using CSs in subclavian artery lesions, especially in total occlusive lesions.

In this study, arterial access was routinely obtained by femoral artery puncture. Brachial access was used in 22 (51.2%) total occlusive patients when the guidewire failed to cross the lesion progradely through the femoral access. The femoral artery approach should be taken as the first candidate for vessel access as far as possible, and the brachial artery approach should be chosen when the femoral artery approach is unavailable or unable to afford a successful intervention. The brachial access is very important for total occlusive lesions. It can not only improve the technical success rate, but also reduce the incidence of complications.

This study has several limitations that are worthy of mention. First, this was a retrospective, nonrandomized study; thus, the choice of using a CS or a BMS was mainly at the surgeon's discretion, leading to inherent biases. Second, the sample size is a little small and there was some loss of follow-up in both groups of patients, which may have affected the final results.

## 5. Conclusions

Interventional therapy has become the primary treatment for SCA occlusive disease because of its minimal invasiveness and safety. The primary patency of CS was significantly higher than that of BMS in left SCA disease. Randomized controlled trials are needed to confirm these results.

## Data Availability

The original contributions presented in the study are included in the article, further inquiries can be directed to the corresponding author.
